# Kidney resident macrophages have distinct subsets and multifunctional roles

**DOI:** 10.1016/j.matbio.2024.02.002

**Published:** 2024-02-07

**Authors:** Christine Chew, Oliver J Brand, Tomohiko Yamamura, Craig Lawless, Mychel Raony Paiva Teixeira Morais, Leo Zeef, I-Hsuan Lin, Gareth Howell, Sylvia Lui, Franziska Lausecker, Christopher Jagger, Tovah N Shaw, Siddharth Krishnan, Flora A McClure, Hayley Bridgeman, Kelly Wemyss, Joanne E Konkel, Tracy Hussell, Rachel Lennon

**Affiliations:** a Lydia Becker Institute for Immunology and Inflammation, Division of Infection, Immunity and Respiratory Medicine, School of Biological Sciences, Faculty of Biology Medicine and Health, The University of Manchester, Manchester M13 9PL, United Kingdom; b Wellcome Centre for Cell-Matrix Research, Division of Cell-Matrix Biology and Regenerative Medicine, School of Biological Sciences, Faculty of Biology Medicine and Health, The University of Manchester, Manchester Academic Health Science Centre, Manchester M13 9PT, United Kingdom; c Bioinformatics Core Facility, Faculty of Biology Medicine and Health, The University of Manchester, Manchester M13 9PL, United Kingdom; d Institute of Immunology and Infection Research, School of Biological Sciences, University of Edinburgh, Ashworth Laboratories, Edinburgh EH9 3FL, United Kingdom; e Department of Paediatric Nephrology, Royal Manchester Children’s Hospital, Manchester University Hospitals NHS Foundation Trust, Manchester Academic Health Science Centre, Manchester M13 9WL, United Kingdom

**Keywords:** Kidney, Macrophages, Basement membrane, Extracellular matrix, Cytometry, RNA-sequencing, Proteomics

## Abstract

**Background:**

The kidney contains distinct glomerular and tubulointerstitial compartments with diverse cell types and extracellular matrix components. The role of immune cells in glomerular environment is crucial for dampening inflammation and maintaining homeostasis. Macrophages are innate immune cells that are influenced by their tissue microenvironment. However, the multifunctional role of kidney macrophages remains unclear.

**Methods:**

Flow and imaging cytometry were used to determine the relative expression of CD81 and CX_3_CR1 (C-X3-C motif chemokine receptor 1) in kidney macrophages. Monocyte replenishment was assessed in *Cx3cr1*^CreER^ X *R26-yfp*-reporter and shielded chimeric mice. Bulk RNA-sequencing and mass spectrometry-based proteomics were performed on isolated kidney macrophages from wild type and *Col4a5*^−/−^ (Alport) mice. RNAscope was used to visualize transcripts and macrophage purity in bulk RNA assessed by CIBERSORTx analyses.

**Results:**

In wild type mice we identified three distinct kidney macrophage subsets using CD81 and CX_3_CR1 and these subsets showed dependence on monocyte replenishment. In addition to their immune function, bulk RNA-sequencing of macrophages showed enrichment of biological processes associated with extracellular matrix. Proteomics identified collagen IV and laminins in kidney macrophages from wild type mice whilst other extracellular matrix proteins including cathepsins, ANXA2 and LAMP2 were enriched in *Col4a5*^−*/*−^ (Alport) mice. A subset of kidney macrophages co-expressed matrix and macrophage transcripts.

**Conclusions:**

We identified CD81 and CX_3_CR1 positive kidney macrophage subsets with distinct dependence for monocyte replenishment. Multiomic analysis demonstrated that these cells have diverse functions that underscore the importance of macrophages in kidney health and disease.

## Introduction

Macrophages display pleiotropic functions depending on their state of activation and immediate tissue microenvironment [1,2]. They are seeded into tissues embryonically and may originate from the fetal yolk-sac or liver, or may be monocyte-derived from adult bone marrow [3]. Embryonic macrophages survive for a considerable time following birth and maintain their numbers through self-renewal [4]. Whether derived from embryonic precursors or the adult bone marrow, tissue resident macrophages play important homeostatic roles in organ health, including: the removal of apoptotic cells and extracellular matrix components, and the promotion of angiogenesis [5,6].

In healthy mice, the kidneys contain a heterogeneous macrophage pool dominated by those derived from yolk-sac progenitors, with recruitment from the periphery increasing with age [7,8]. Replenishment of macrophages from their precursors is observed in most inflamed tissues, and are likely to be directed by the niche in which they enter [9, 10]. Aged kidney macrophages display reduced pro-inflammatory responses, which may be due to changes in local mesenchymal stromal cells [11]. Peripheral influences can also affect kidney macrophages by increasing their numbers and pre-priming them for subsequent insults [12].

The notion of tissue-specific macrophages has recently been refined by considering their position within the local milieu. Rather than an organ-specific population, macrophages modify their transcriptional expression and responses within a particular tissue niche. For example, SpiC and LXRa define the red pulp [13] and marginal zone [14] splenic macrophages, respectively. Also, discrete populations such as perivascular and meningeal macrophages co-exist in the brain alongside microglia, each performing very different roles [15]. Similarly, kidney macrophage populations exhibit distinct phenotypes depending on whether they reside in the medulla or cortex, and activation is likely to be influenced by variability in cell signaling within the organ [16]. Commonalities may therefore exist between macrophages in different organs because of their specific niche or function required within that distinct environment [17].

The divergent roles of resident and recruited macrophages have led to intense investigation of macrophage heterogeneity [9] with the aim of identifying new therapeutics targeting specific pathogenic subsets. Recently, CD74 (macrophage migration inhibitory factor receptor) and CD81 (tetraspanin family) were proposed as cell surface markers for kidney resident macrophages [18]. Similar to previous studies, though using different markers [19,20], peripheral blood monocytes minimally contributed to resident CD74/CD81^+^ macrophages at steady state [18]. Another marker widely used to distinguish monocyte-replenished and resident macrophage subsets is the chemokine receptor CX_3_CR1 (fractalkine receptor), important for monocyte survival that identifies resident macrophages in the gut [21,22], brain [23] and kidneys [24,25].

In this study, using CD81 and CX_3_CR1 as macrophage markers, we define three kidney macrophage subsets and their different requirement for monocyte replenishment. With RNA-sequencing and proteomics we identify distinct biological functions, suggesting multifunctional roles of kidney macrophages.

## Results

### Kidney macrophage subsets are identified by the markers CX_3_CR1 and CD81

**Fig. 1:**
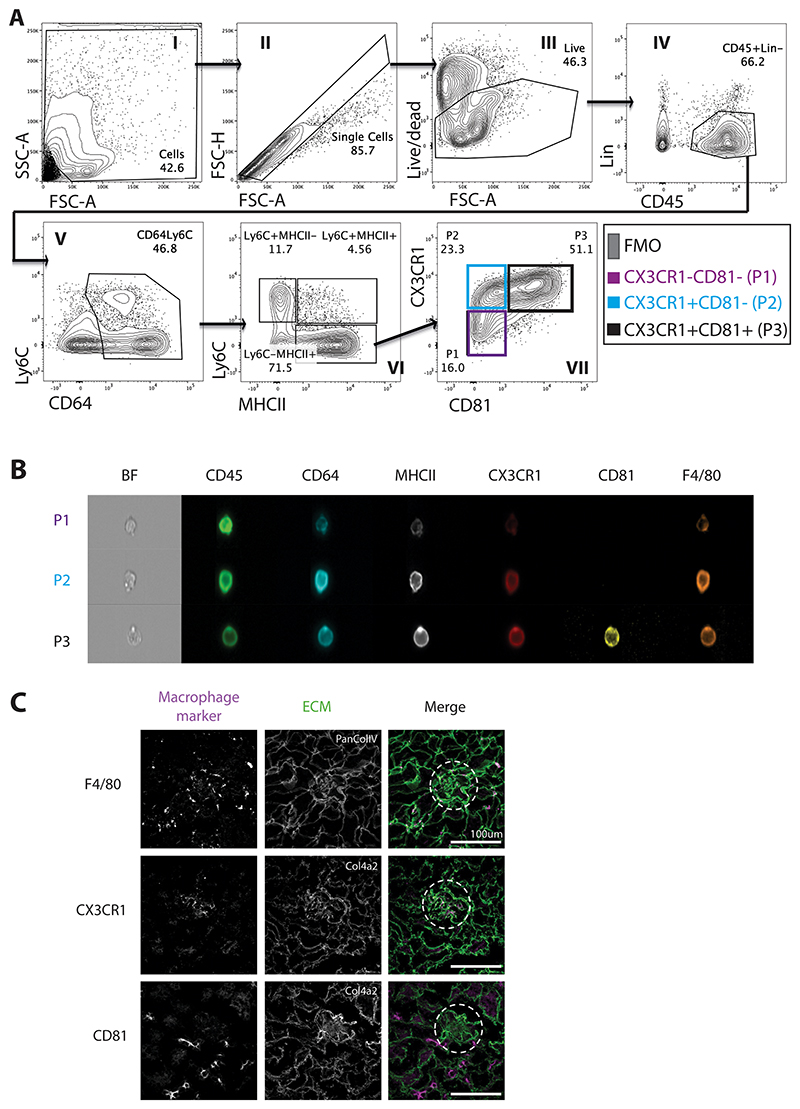
CX_3_CR1 and CD81 identify three kidney macrophage subsets. a, Representative flow cytometry contour plots showing the gating strategy for identifying kidney macrophage subsets defined by CX_3_CR1 and CD81. Cells were identified as (I) Side scatter area (SSC-A) versus forward scatter area (FSC-A), (II) forward scatter height (FSC–H) versus FSC-A to exclude cell doublets, (III) Live/dead versus FSC-A to identify live or viable cells (IV) Lineage (Lin: CD3, CD19 and Ly6G) versus CD45 to identify hematopoietic populations and exclude lymphoid and neutrophil populations, (V) Ly6C versus CD64 to identify monocytes and macrophages, (VI) Ly6C versus MHCII to identify Ly6C^+^MHCII^−^ monocytes, Ly6C^+^MHCII^+^ monocyte intermediates and the Ly6C^−^MHCII^+^ macrophage pool, (VII) CX_3_CR1 versus CD81 to identify three macrophage subsets from Ly6C^−^MHCII^+^cells, including CX_3_CR1^−^CD81^−^ (P1: purple), CX_3_CR1^+^CD81^−^ (P2: blue) and CX_3_CR1^+^CD81^+^ (P3: black). Flow plots are representative of at least ten independent experiments. b, Imaging cytometry of kidney macrophage subsets defined by CX_3_CR1 and CD81 in C57BL/6 mice at steady state. Kidney single cell suspensions were incubated with antibodies for a viability dye, CD45, lineage (CD3, CD19, Gr-1), CD64, MHCII, CX_3_CR1, CD81 and F4/80 antibodies and macrophages identified based on the expression of these markers, which include the P1, P2 and P3 kidney macrophage subsets. c, Immunofluorescence of F4/80, CX_3_CR1 and CD81 (macrophage markers, purple), pan-collagen IV (PanColIV) and Col4a2 (basement membrane markers, green) in kidney sections (3 μm thick) from C57BL/6 mice at steady state. Dashed circle: glomerulus. Merge: White scale bar, 100 μm. Images are representative of >2 independent experiments.

To unravel the heterogeneity of kidney macrophages, we examined their segregation using the markers CX_3_CR1 and CD81 and compared subsets to widely used markers CD11b and F4/80 that conventionally distinguish monocyte-derived (CD11b^high^F4/80^low^, MP1) and tissue resident (CD11b^low^F4/80^high^, MP2) macrophages [26]. Both resident and monocyte-derived macrophages expressed CX_3_CR1 and CD74, but only the resident MP2 macrophages strongly expressed CD81 (Supplementary Figure S1A). We identified the kidney monocyte/macrophage pool through the established “monocyte waterfall” model of monocyte/macrophage differentiation (Fig. 1A), in which Ly6C^+^MHCII^+^ cells act as developmental intermediates, connecting the Ly6C^+^MHCII^−^ monocytes and Ly6C^−^MHCII^+^ macrophages [27]. Co-expression of CX_3_CR1 and CD81 was predominantly observed in the Ly6C^−^MHCII^+^ macrophage pool (Supplementary Figure S1B). Within this macrophage pool, we identified three populations: CX_3_CR1^−^CD81^−^, CX_3_CR1^+^CD81^−^ and CX_3_CR1^+^CD81^+^, which we called: P1, P2, and P3, respectively. By backgating these subsets onto a CD11b and F4/80 flow plot (Supplementary Figure S1C), we deduced that P1 is similar to monocyte-derived MP1 macrophages, P2 contains both MP1 and MP2 but appears to be slightly MP2-dominant and P3 is comparable to tissue resident MP2 macrophages. We compared P1, P2 and P3 kidney macrophage subsets to populations identified using conventional markers CD11b and F4/80 in previous publications (Supplementary Table S1) and found overlap in marker expression.

We next examined the morphology of the kidney macrophage subsets defined by CX_3_CR1 and CD81 using imaging cytometry. The P1 population appeared smaller than the CX_3_CR1^+^ subsets (P2 and P3) (Fig. 1B). All macrophage subsets expressed CD45, CD64 and MHCII, demonstrated here in histograms (Supplementary Figure S1D). Consistent with the flow cytometry data, CD81 was restricted to P3 macrophages. We then examined the location of different kidney macrophage populations by immunofluorescence (Fig. 1C, Supplementary Figure S1E). F4/80, CX_3_CR1 and CD81 signals were observed in the peri-glomerular region. The peri-glomerular area is known to harbour kidney macrophages [19]. Together, these findings demonstrate that the macrophage subsets identified by CX_3_CR1 and CD81 are consistent with the cellular morphology and localization expected of kidney macrophages.

### Kidney macrophage subsets differ in their requirement for monocyte replacement

**Fig. 2:**
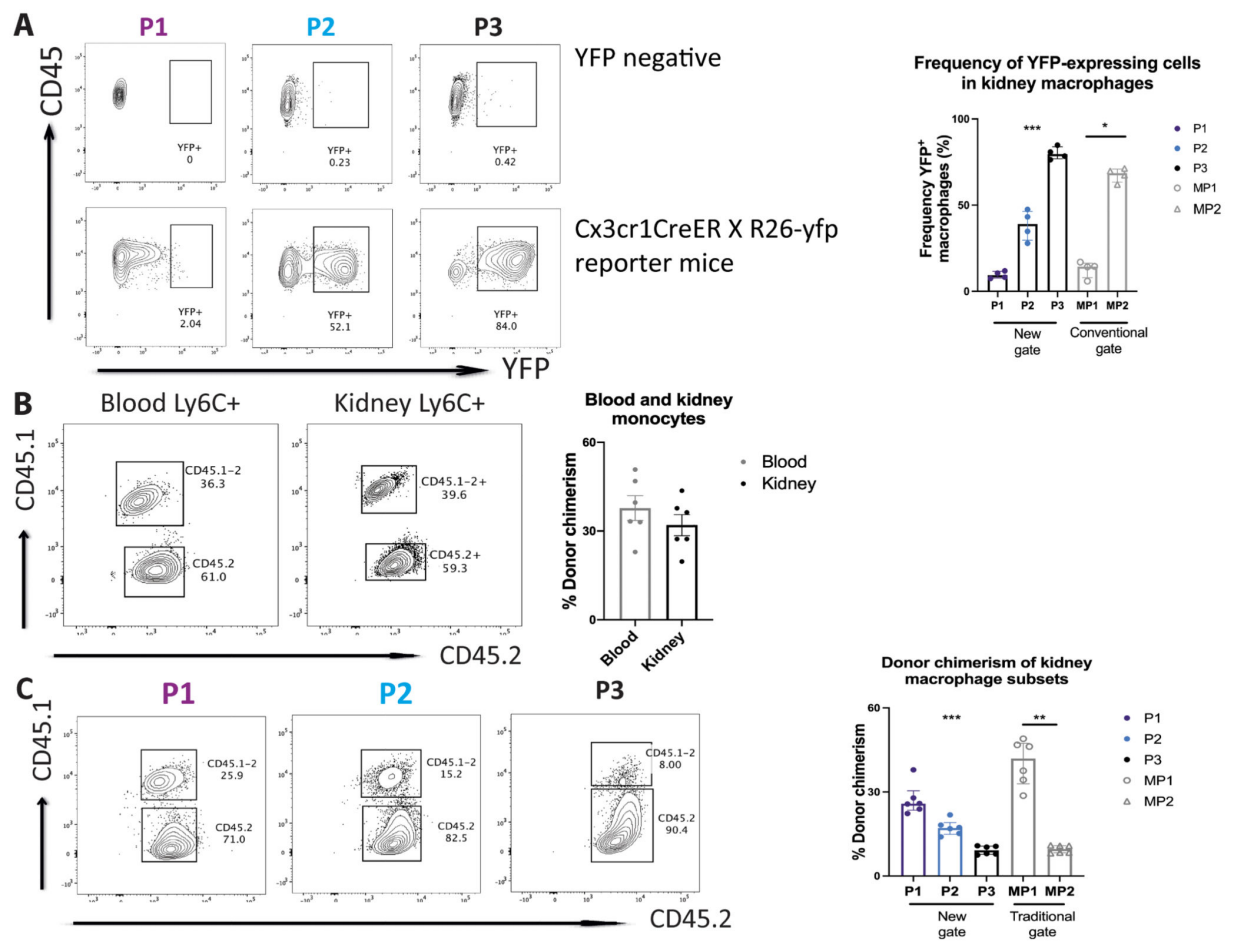
CX_3_CR1 and CD81 identify kidney macrophages with distinct monocyte replenishment. a, *Cx3cr1*^CreER^ X *R26-yfp* reporter mouse model and the assessment of macrophage replenishment by blood monocytes represented by YFP^−^ cells 16 weeks after tamoxifen treatment. Representative flow cytometry contour plots (left) to assess YFP expression in P1, P2 and P3 kidney macrophage subsets compared to a YFP-negative control. Graph (right) showing frequencies of YFP-expressing macrophage subsets defined by the new (P1, P2, P3) and conventional (MP1, MP2) gate. b, From the blood and kidneys of shielded chimeras at 7 weeks after irradiation and reconstitution with CD45.1/2 donor bone-marrow. Representative flow cytometry contour plots (left) and graph (right) showing the percentage (%) of chimerism for the blood and kidney Ly6C^+^monocytes of shielded chimeras. c, Representative flow cytometry contour plots (left) showing the percentage of chimerism for P1, P2 and P3 kidney macrophages subsets. Graph (right) showing percentage of chimerism of kidney macrophage subsets defined by the new (P1, P2 and P3) and conventional (MP1, MP2) gate. Data are representative of two independent experiments. Graphs show the median (IQR) of *n* = 4–6 mice per group. Multiple comparisons between P1, P2 and P3 macrophages were performed with a Kruskal-Wallis test and statistical comparisons between MP1 and MP2 were performed with a Mann-Whitney U test, *p<0.05, **p<0.01, ***p<0.001.

Whilst the ontogeny of CD11b and F4/80-expressing kidney macrophages has been reported previously [20,28], the replenishment of subsets defined by CX_3_CR1 and/or CD81 have not been described. To explore kidney macrophage ontogeny, we crossed *Cx3cr1*^CreER^ with *R26-yfp* animals and administered tamoxifen to induce the nuclear translocation of the cre enzyme and irreversible yellow fluorescent protein (YFP) expression in CX_3_CR1^+^ cells as described previously [24, 29,30], strongly labelling kidney macrophages. Cells replenished from blood monocytes after tamoxifen withdrawal will express cre in the cytoplasm, thus identify as YFP^−^cells [30]. Mice were left for 1 week (Supplementary Figure S2A) or 16 weeks (Fig. 2A) after the final tamoxifen dose to assess kidney macrophage replenishment by blood monocytes. In the kidneys of YFP-reporter mice at 16 weeks, replenishment of P3 macrophages was lower than in P2, whereas there was increased replacement of P1 macrophages from YFP^−^ monocytes. When compared to conventionally gated macrophage subsets, the proportion of YFP^−^ monocytes required to replenish P3 was similar to the resident MP2 subset.

Next, we verified the monocyte replacement of macrophages using a shielded bone marrow approach as previously described [30]. Recipient mice (CD45.2) were irradiated with the abdomen shielded (Supplementary Figure S2B). Shielded irradiation maintains the niche and enables steady state replenishment of macrophages to be determined. Mice were then reconstituted with donor CD45.1/2 bone marrow cells and the blood and kidneys examined seven weeks post-procedure. In both non-irradiated controls and radiated animals, the total number of kidney macrophages and respective subsets was similar (Supplementary Figure S2C), indicating that shielding had prevented prolonged tissue perturbation in these animals.

The chimerism of blood and kidney Ly6C^+^ monocytes with donor CD45.1/2-expressing cells was similar (Fig. 2B). The degree of chimerism was different between P1, P2 and P3 kidney macrophages (Fig. 2C). The P1 subset had the highest chimerism compared to P2 and P3, indicating predominant replenishment from blood monocytes. P3 macrophages, equivalent to tissue resident MP2 macrophages, had the lowest chimerism, suggesting the least reliance on blood monocytes for their local maintenance. The pattern of monocyte replenishment in shielded chimeras and YFP-reporter mice was similar. The P3 subset had comparable chimerism to kidney resident MP2 macrophages and similarly shielded models (Supplementary Figure S2D) [31]. Therefore, using two independent models, we found that macrophage subsets defined by CX_3_CR1 and CD81 are distinct in the requirement for replacement from blood monocytes. These data confirm CD81 as an underappreciated marker for the kidney resident macrophage population.

### RNA-sequencing of kidney macrophages

**Fig. 3:**
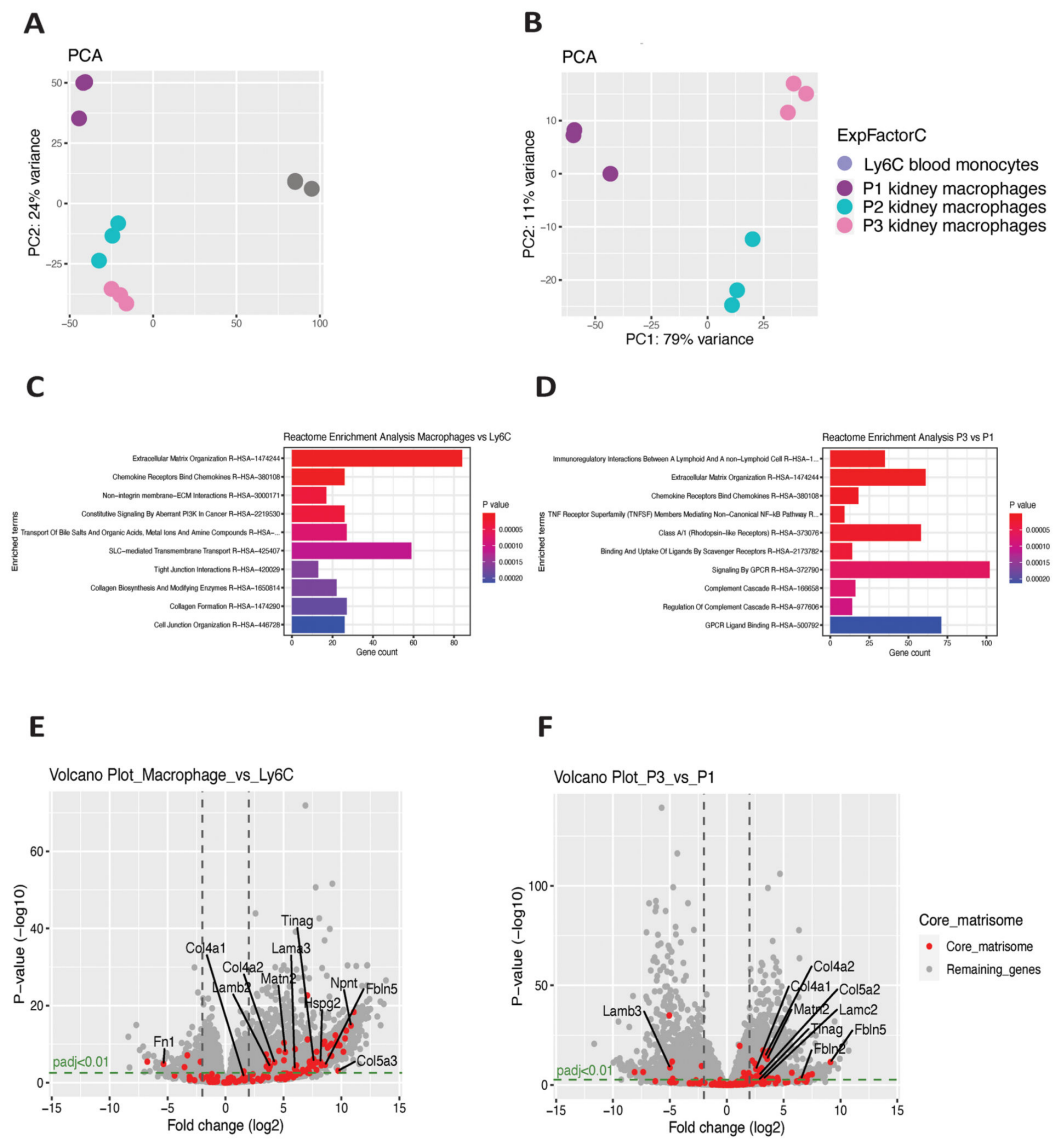
Kidney macrophage subsets defined by CX_3_CR1 and CD81 are transcriptionally distinct and express transcriptomic profiles consistent with matrix related function. Bulk RNA-sequencing data of P1, P2 and P3 kidney macrophage subsets and Ly6C^+^ blood monocytes isolated by FACS represented by a, principal component analyses (PCA) of global gene expression from P1 (purple), P2 (blue) and P3 (pink) kidney macrophage subsets and Ly6C^+^monocytes (grey). b, PCA of global gene expression from P1, P2 and P3 kidney macrophage subsets. Selected pathways from the reactome enrichment analysis of transcriptomic datasets from bulk RNA-sequencing from c, kidney macrophages versus Ly6C^+^ monocytes and d, P3 versus P1 kidney macrophages. Transcript expression profile (volcano plot) of: e, kidney macrophages versus Ly6C^+^ monocytes and f, P3 versus P1 kidney macrophages. Dashed lines represent an adjusted p value <0.01. Data are representative of three independent cell sorts from the kidneys and blood of 4–5 pooled C57BL/6 mice at steady state.

To further explore the heterogeneity of the kidney macrophage pool, we sorted the P1, P2 and P3 subsets alongside their circulating Ly6C^+^ blood precursor by FACS and performed bulk RNA-sequencing. Principal component analysis (PCA) on three experimental replicates revealed that the three macrophage subsets were transcriptionally distinct from blood monocytes (Fig. 3A) and from each other (Fig. 3B). The CX_3_CR1^+^ subsets (P2 and P3) were more closely related to each other than with P1.

Hierarchical clustering of differentially expressed genes confirmed that blood monocytes and kidney macrophages are distinct (Supplementary Figure S3A). Differentially expressed genes (log_2_ fold change (log_2_FC)>2, FDR adjusted *p*<0.01) upregulated in the P3 subset included *Mertk, Axl, Mrc1, Fcgr1, Cx3cr1, Adgre1, C1qa-c, Cd74* and *Cd81* consistent with the transcriptional profile of mature resident macrophages in other tissues [32] and kidney macrophages [5,18,33]. Flow cytometry analysis detected CD64 (Supplementary Figure S3B), CD74 and CD81 (Supplementary Figure S1A) in kidney macrophages, but unlike in lung macrophages, “macrophage-associated markers” [32] such as MerTK and Axl were not detected (Supplementary Figure S3B). Together, the transcriptional profile of P3 kidney macrophages conform to transcripts expressed by resident macrophages from other organs but variation in protein detection by flow cytometry exists, highlighting the diversity of macrophage characterization across tissues.

To determine the relevance of differentially expressed genes from our RNA-sequencing dataset, we performed functional enrichment analyses (adjusted *p*<0.01). In addition to immune related functions, macrophage responses in the kidney may be regulated by extracellular matrix components. When comparing macrophages against Ly6C^+^ monocytes (Fig. 3C), one of the key pathways highlighted in our reactome enrichment analysis included extracellular matrix terms. In our functional reactome enrichment analysis, we compared macrophages to each other (Fig. 3D, Supplementary Figure S3C), which highlighted extracellular matrix terms in addition to known macrophage functions including chemokine receptor binding, complement cascade regulation and TNF-receptor superfamily mediation of the NF-κB pathway. The inclusion of these matrix related pathways is unexpected in kidney macrophages at steady state. While it is known that macrophages regulate innate immunity (Supplementary Table S2A-C) and signal to other cells involved in matrix deposition during disease [34], the expression of matrix associated genes warranted further investigation.

Next, we unified the transcripts associated with matrix related pathways using a curated list of matrisome genes [35]. We found 2756 upregulated transcripts and 219 downregulated transcripts in kidney macrophages relative to Ly6C^+^ blood monocytes (adjusted *p*<0.01, log_2_FC>2) (Fig. 3E). This included upregulation of type IV and V collagens (*Col4a1, Col4a2, Col5a3*), laminins (*Lama3, Lamb2*) and other matrix transcripts (*Matn2, Hpsg, Npnt, Fbln5, Tinag*). We also directly compared transcript expression in P3 and P1 macrophages (Fig. 3F). This analysis indicated distinct transcript expression profiles between the two macrophage subsets without using monocytes as a comparator. Again, we observed upregulation of transcripts similar to our previous comparison of macrophages with monocytes, which included *Lamc2* and Fbln2, and downregulation of *Lamb3*. This indicates that the matrix transcript profile was maintained in macrophages subsets without using monocytes as a relative comparison. We also normalized gene counts by rpkm (reads per kilobase per million mapped reads) to enable quantitative comparisons of expression levels within our dataset (Supplementary Table S3a), which highlighted matrix-related transcripts. Together, our data implies that macrophages defined by CX_3_CR1 and CD81 may possess a matrix transcriptional profile at steady state within the kidney microenvironment.

With all cell purification strategies, contamination by other cell types is possible. Despite our best efforts in using a gating strategy to identify cells that most represent macrophages, there are limitations in that current and available markers may not enable the exclusive selection of macrophages. We compared isolated macrophage subsets (P1–3) with a bulk RNA-sequencing dataset from 3 month old mouse kidneys [36]. This comparison revealed expression of stroma (*Col3a1*), tubule (*Umod, Slc12a1, Lrp2, Maf, Aqp2* and *Atp6v1g3*) and podocyte (*Nphs2* and *Podxl*) related transcripts, in P1, P2 and P3 subsets but not Ly6C^+^ monocytes (Supplementary Figure S4a), indicating some degree of non-immune cell contamination. We also detected that *C1qb* is highly expressed across all scatterplots, indicating the presence of macrophages. To understand the extent of the contamination, we used CIBERSORTx [37] to estimate the proportion of immune and non-immune cell types in our bulk RNA-sequencing data. We built a mouse kidney signature matrix containing 16 cell types from published scRNA-sequencing datasets including GSE128993 (CD45^+^ innate immune cells from whole kidney, *n* = 1) [18], GSE140023 (renal cortex, *n* = 4) [6], GSE174324 (CD11b^+^F4/80^+^ cells from whole kidney, *n* = 1) [38] and GSE193892 (CD11b^+^F4/80^+^ cells from whole kidney, *n* = 4) [25] to estimate the relative fractions of 16 immune and non-immune cell types in bulk tissue transcriptomes (Supplementary Table S3b, Supplementary Figure S4b, c). This analysis showed that the P1 subset contained a low proportion (~6 %) of macrophages in contrast to Ly6C^+^ blood monocytes (92 %). The P2 subset was more enriched with 15 % infiltrating and 29 % resident macrophages, and the P3 subset had around 74 % resident macrophages but no infiltrating macrophages. The presence of podocyte transcripts is potentially a source of contamination within the analyses (Supplementary Figure S4a) but this may also highlight the close association of macrophages with other cell types.

### Extracellular matrix proteins are detected in kidney macrophages

**Fig. 4:**
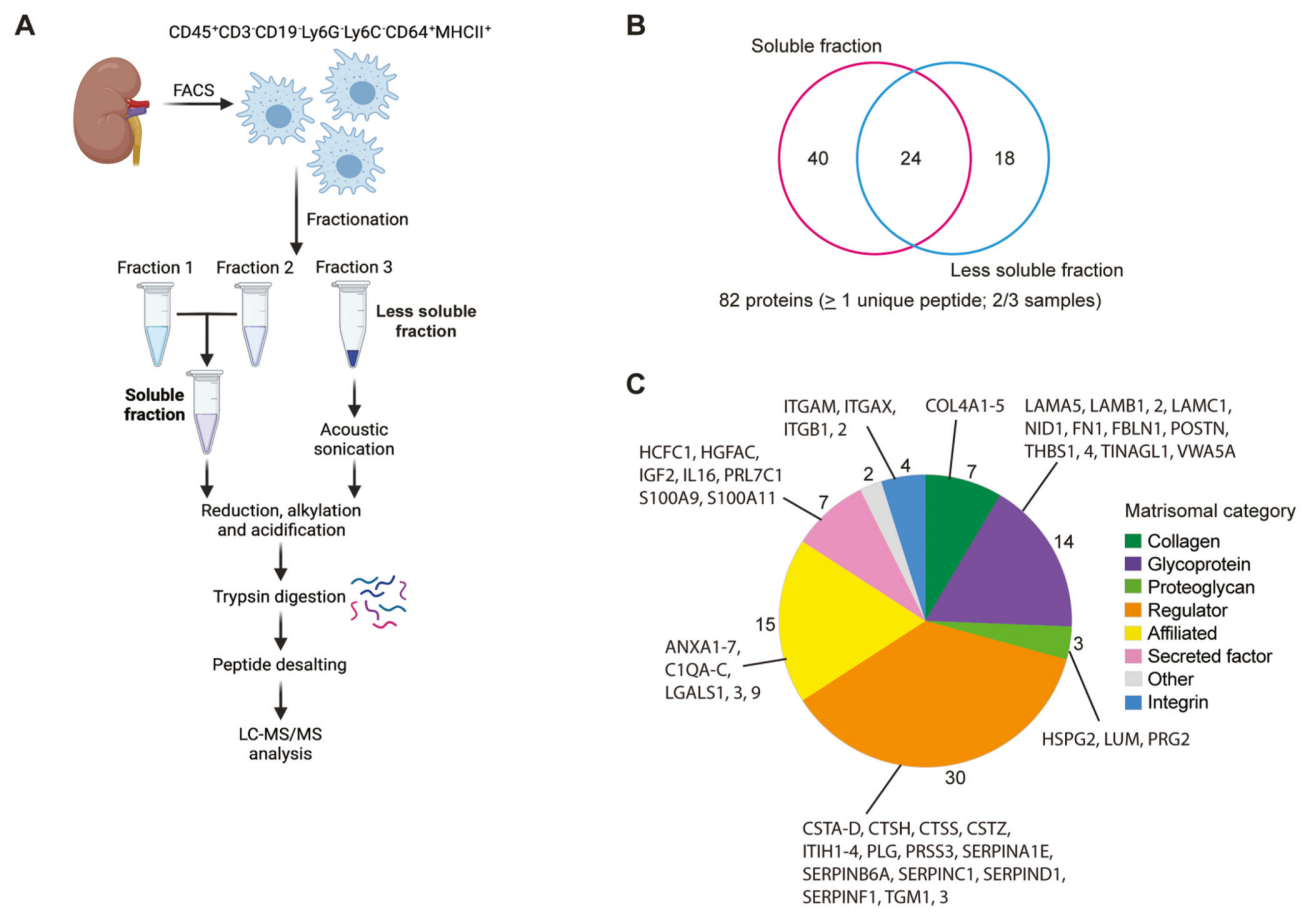
Proteomic analyses of kidney macrophages identify pathways associated with basement membrane homeostasis. a, Workflow showing sample enrichment for ECM proteins of the isolated kidney macrophage pool. b, Soluble and less soluble fractions prepared from the kidney macrophage pool (*n* = 3) analyzed by mass spectrometry and the overlap for ECM proteins represented by a Venn diagram. c, Proportion of proteins expressed by the kidney macrophage pool according to the matrisomal category represented by a pie chart. Data are representative of three independent sorts from the kidneys of 6–8 pooled C57BL/6 mice at steady state for proteomic analyses.

All prior analyses relate to RNA transcripts. We therefore next determined whether matrix proteins could be detected in our enriched kidney macrophage subsets. For this, we performed matrix enrichment [39,40], in sorted macrophage pools (Supplementary Figure S5A), which yielded two fractions, a soluble cellular fraction and a less soluble matrix-enriched fraction, which we analyzed by mass spectrometry-based proteomics (Fig. 4A). Overall, we identified 1843 proteins (see Supplementary Methods; Supplementary Figure S5B, Supplementary table S4A, B), and with functional enrichment analyses (see Methods) we found associations with macrophage function, including cellular response to cytokine stimulus, antigen processing and presentation, and regulation of phagocytosis (Supplementary Figure S5C, Supplementary table S5A, B). In addition to pathways relating to immune function, we found extracellular matrix terms such as cell adhesion regulation. We then focussed on pathways that are integral to matrix homeostasis.

**Fig. 5:**
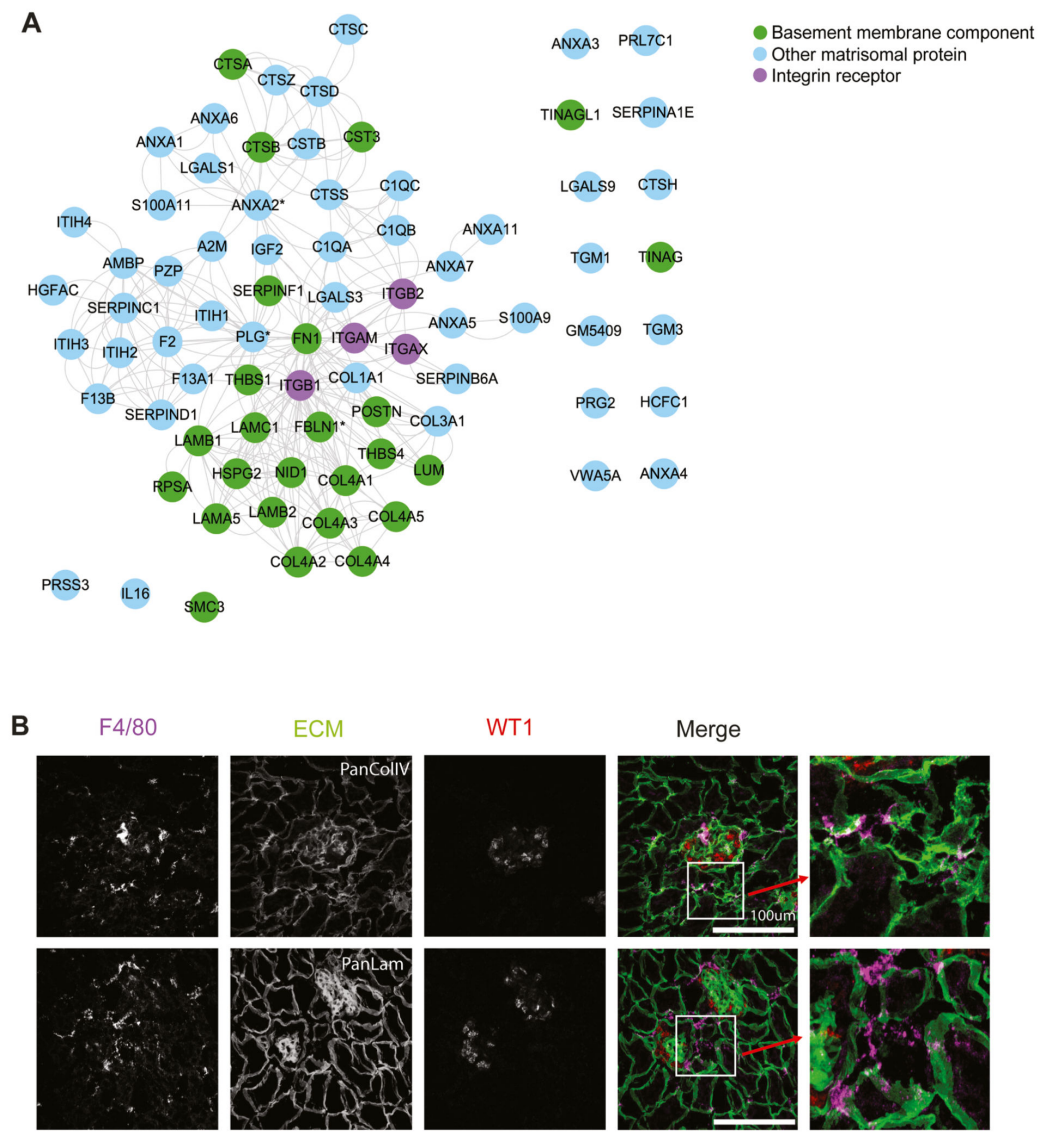
ECM components are detected in kidney macrophages. a, Protein interaction network showing basement membrane components, other ECM proteins and integrin receptors identified in kidney macrophages (nodes represent proteins and connecting lines indicate protein-protein interaction). Data are representative of three independent sorts from the kidneys of 6–8 pooled C57BL/6 mice at steady state for proteomic analyses. b, Immunofluorescence of F4/80 (purple), pan-collagen IV (PanColIV) and pan-laminin (PanLam) (basement membrane markers, green) and WT1 (red) in kidney sections measuring 3 μm from C57BL/6 mice at steady state. Merge: White scale bar, 100 μm. Images are representative of >2 independent experiments.

We identified 82 matrix proteins in the proteomic analysis (Supplementary table S4C, Fig. 4B). These included core basement membrane collagen IV chains (COL4A1–5), glycoproteins (LAMA5, LAMB1/B2, LAMC1, NID1), matrix regulators such as cathepsins and protease inhibitors, and matrix-affiliated proteins including annexins and galectins (Fig. 4C, 5A). We also detected four integrin receptors ITGAM/X and ITGB1/2 (Fig. 5A), which are complement receptor and markers of monocyte/macrophage adhesive interactions. In the soluble fraction, we found RRBP1 [41] and VIM [42], which are known to interact with collagens. Additionally, we identified FERMT3, TLN1, LGALS3, HRNRPH1 and ILK, which are integrin associated proteins. We also found C1QA-C protein expression (Supplementary table S4B, Fig. 5A), confirming the presence of kidney macrophages. In the soluble fraction, we detected GPX1 and CRIP1, key matrix proteins relating to macrophage tissue residence which have also been described in single-cell kidney macrophage data [18]. With immunofluorescence we detected collagen IV colocalization with F4/80^+^ macrophages within glomerular structures (Fig. 5B).

### Kidney macrophage matrix profiles and localization in Alport mice

**Fig. 6:**
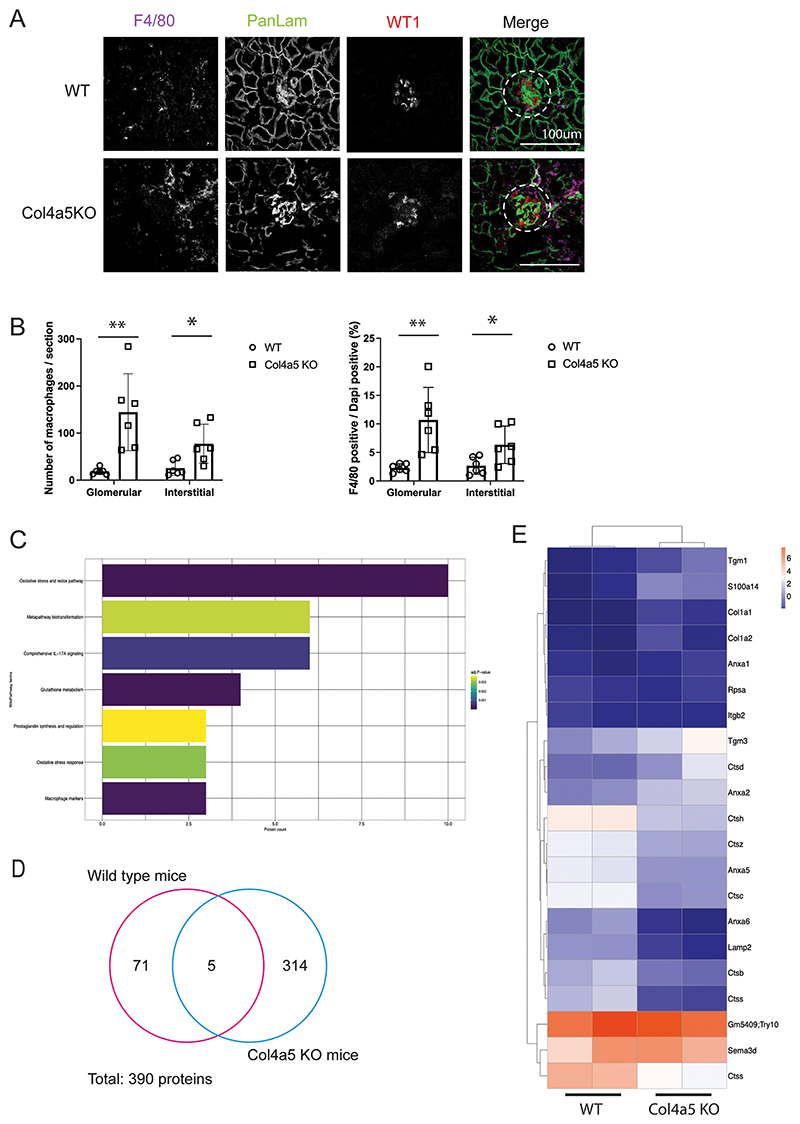
ECM components in *Col4a5*^−*/*−^ mice with a basement membrane defect. a, Immunofluorescence of F4/80 (purple), pan-collagen IV (PanColIV) (green) and WT1 (red) in kidney sections (3 μm thick) from wild type and *Col4a5*^−*/*−^ (Col4a5KO) mice. Dashed circle: glomerulus. Merge: White scale bar, 100 μm. **b**, Graph showing the number of glomerular and interstitial macrophages per section (left) and proportion of F4/80-positive/DAPI-positive cells (right) in wild type and *Col4a5*^−*/*−^ mice (Col4a5 KO). Images are representative of >2 independent experiments. Statistical comparisons between macrophages in different groups were performed with a Mann-Whitney U test, *p<0.05, **p<0.01 **c**, Graph depicting selected functional enrichment pathways of proteomic datasets from mass spectrometry analyses showing biological processes of purified macrophages from wild type and *Col4a5*^−*/*−^ mice with the protein count (X-axis) that have highlighted ontology annotations. Biological WikiPathway terms were considered enriched when adjusted *p*<0.001. **d**, Soluble fractions prepared from a kidney macrophage pool analyzed by mass spectrometry and the overlapping proteins represented in a Venn diagram. **e**, Heatmap of extracellular matrix (ECM)-related protein expression profile of purified kidney macrophages from wild type and *Col4a5*^−*/*−^ mice. Data are representative of macrophages from the kidneys of 6–8 pooled wild type and *Col4a5*^−*/*−^ mice at steady state for proteomic analyses.

Next, we examined *Col4a5*^−*/*−^ (Alport) mice, which have a primary defect in basement membrane assembly [43]. We found an increased signal for F4/80 in the kidney sections of *Col4a5*^−*/*−^ mice examined by immunofluorescence, compared to wild type mice (Fig. 6A, B). We performed a proteomic analysis of isolated *Col4a5*^−*/*−^ kidney macrophages and from the cellular fraction we found enrichment of biological pathways related to macrophage function including comprehensive IL-17A signalling, prostaglandin synthesis and regulation, oxidative stress response and macrophage markers (Fig. 6C). Overall we identified 390 proteins in the proteomic analyses (Fig. 6D), including components associated with matrix homeostasis (ANXA5, ANXA6 and LAMP2) and interstitial matrix components (COL1A1/2) (Fig. 6E, Supplementary table S6). We detected CTSC, CTSH, CTSS and CTSZ, which have a role in MHC class II molecule antigen presenting function and activation. We also identified S100A14, which is known to promote cell motility and invasiveness through regulating the matrix metalloproteinase 2 (MMP2) function and expression [44]. S100A14 is also detected in scRNA-sequencing cluster expression profiles in mice with late kidney injury [6]. Additionally, the detection of proteins such as LAMP1 in Alport macrophages may indicate inflammation [45]. Finally we used RNAscope to determine whether there was colocalization of matrix transcripts and macrophage markers. We found an overlap in the signals for F4/80 and Col4a1, indicative of co-expression in a subset of macrophages (Supplementary Figure S5D). It is plausible that matrix production by Alport macrophages is triggered by the injury environment.

In the isolated macrophage pool used for proteomic data, we did not find evidence of contamination by podocytes, endothelial and mesangial cells, but found some markers of fibroblasts and tubular cells (Supplementary Table S7a, b). The fibroblast markers detected in the data were extracellular matrix components (collagen I and III, fibronectin and lumican) and the tubular markers were Lrp2, Ass1 and Gpx3, where the latter is also expressed in mouse kidney macrophages [46].

## Discussion

We have shown heterogeneity in kidney resident macrophages and that the marker CD81 delineates unappreciated subsets that have distinct reliance on monocyte replenishment. With multiomic analysis we identified multiple enriched biological processes, including extra-cellular matrix organisation and, this was supported by the colocalization of Col4a1 with F4/80 in a subset of kidney macrophages.

We utilized CD81 and CX_3_CR1 and identified three previously undescribed kidney macrophage subsets. The scRNA-seq analyses of CD45^+^ cells resolved CD81 not only in mouse resident kidney macrophages, but also across several species [18], enabling the translation from animal models to human biology and disease [47]. The choice of CD81 over CD74 was based on bulk RNA-sequencing validation data of monocyte-derived and resident kidney macrophages, showing *Cd81* as one of the top differentially expressed genes between these subsets that matched scRNA-seq data [18]. The first description of canonical kidney monocyte/macrophage populations utilised CX_3_CR1 [48], which also identifies tissue resident macrophages in the gut [21,22], and brain [23]. Consistent with the expected profile of resident macrophages, the gene profiles of all kidney macrophage subsets in our RNA-sequencing dataset were associated with biological pathways related to immune system functions. The transcriptional profiles displayed by kidney macrophage subsets were consistent with previously published datasets on tissue resident macrophages across organs [32], which included *MerTK, Fcgr1, Axl* and *Mrc1* amongst others. The genes with increased expression in the P2 and P3 kidney macrophage subsets such as *Adgre1, Apoe* and *Mrc1*, and complement-related transcripts (*C1qa-c*) agree with previous studies [18,49,50]. However, the expression of gene transcripts did not match flow cytometry validation of some of these *bona fide* markers including Axl and MerTK in our comparison of kidney and lung macrophages. The differences in the expression of myeloid population markers may be due to tissue dissociation techniques or cell activation state, which should be considered given the focus of our data on steady state. Nevertheless, myeloid marker expression is known to vary across tissue macrophages, reflecting the need for more kidney-specific candidates.

We have demonstrated that kidney macrophage subsets could be distinguished based on their dependence on circulating monocytes for turnover by using the YFP-reporter and shielded chimeric mice. Differences in monocyte replacement of kidney macrophages exist [5,28,33, 51], which may be attributed to variability in the models used, sex and housing environment [31]. In shielded chimeras, monocyte replacement of F4/80^+^ kidney macrophages is proportionally higher in males compared to females on a C57BL/6 background [31]. In our experiments in the YFP-reporter model, there were no differences in monocyte replenishment of kidney macrophages between sexes. For phenotypic consistency, shielded chimeras only included males, which have a higher level of chimerism compared to females in the kidneys [31]. In our data, the percentage of chimerism in MP2 resident macrophages, comparable to the P3 subset, was higher than previously reported in parabiotic mice [20,28], but agreed with levels observed from tissue-protected animals [31]. The chimerism of P3 macrophages was higher than levels shown by other tissue resident macrophages including the microglia, alveolar macrophages, Langerhans cells and splenic macrophages [31]. Our data indicate that P3 macrophages had a partial dependence on monocyte replacement for turnover at steady state.

We were surprised to find RNA transcripts for matrix components in kidney macrophages. The extracellular matrix is a complex polymer network of proteins that form interstitial matrix and basement membrane structures and include components such as collagen IV and laminins [43]. Basement membranes appeared at the dawn of multicellularity [52], and these structures are key determinants of developmental processes that are necessary for tissue formation [53]. Challenging the established paradigm that basement membranes are static matrices [54], there is increasing evidence that these structures turnover [55–59] and are maintained by matrix components that regulate developing organs in a tissue-specific manner [60–62]. Most studies profiling matrix component production by macrophages have focused on diseased states such as cancer [63,64] or cell-based studies [65]. Recent scRNA-seq analyses have shown enrichment of matrix transcripts by kidney macrophages in disease [6]. However, whether the kidney-specific environment influences matrix component expression by mammalian macrophages at steady state in vivo is largely unknown. Intriguingly, we found that in addition to the expected transcriptional profile relating to immune function, matrix transcripts such as collagen IV and laminins were expressed by the P2 and P3 kidney macrophage subsets at steady state. Functional enrichment analyses of RNA-sequencing datasets revealed that P2 and P3 macrophages had a more distinctive matrix transcriptional profile compared to P1.

However, there are several limitations to our study. Firstly, purification of macrophages is technically very difficult as the markers used commonly overlap with other cell types and we are restricted to commercially available fluorescent antibodies and surface markers usually identified through scRNA-sequencing [18]. By using CIBER-SORTx [37] (a bioinformatics tool used to deconvolute cell type proportions from bulk RNA sequencing datasets) we found potential contamination of our three kidney macrophage populations with other cell types. This contamination included podocytes, which have been described to express immune cell markers and function as previously reviewed [66,67]. Conventional dendritic cells and a small signal from fibroblasts were also present. Intriguingly, this may indicate cells that macrophages are interacting with in the kidney microenvironment. Where strong interpretations have been made in the past based on total RNAseq data, it would be interesting to apply CIBERSORTx to these datasets to assess the purity of the sample.

Secondly, there is a possibility that despite an increased fold expression in matrix-related transcripts, this may have arisen from a low baseline or from biological variability between samples. Unlike RNAseq however, we could not detect any signs of cell contamination in our proteomic dataset, which may be due to the measurement of peptides by abundance. Thirdly, macrophages a relatively small population of cells amongst other cell types in the kidneys, constituting around 5 % of the whole kidney cell population of 50 000 cells from healthy mice [49]. Genes expressed in smaller cell populations within a larger pool of other cell types can still have functional significance. Despite the population of macrophages being smaller compared to other cell types, they can exert significant homeostatic effects within their immediate tissue environment in response to inflammatory stimuli [68].

That matrix associated pathways were present in transcriptomic and proteomic datasets too remains surprising. It was not experimentally possible to purify enough viable macrophages from the kidney for functional analysis; ideally to examine matrix secretion. There would remain the caveat that any production could reflect expulsion of matrix engulfed during the assay. The closest we have to proof of matrix expression is the RNAscope analysis, which shows co-localisation of F4/80 and Col4a1 transcripts in situ. Even this however is technically challenging due to the small subset of macrophages identified.

While we were limited to the total macrophage pool for proteomic analyses, this system provided the closest representative to the specific cell type in vivo without losing cellular material and avoided significant contamination from non-myeloid cell populations. It was not possible to determine proteomic profiles of specific macrophage subsets due to the low yield of cells and the number of animals required for these experiments. The pathways highlighted in this proteomic dataset aligned with the transcriptional profiles of the segregated macrophage subsets, including pathways associated with matrix regulation. In *Col4a5*^−*/*−^ mice, the proteins we identified from kidney macrophages included S100A14, LAMP2 and ANXA2, which indicate possible compensation for altered basement membrane composition, however this finding requires further interrogation. Our hypothesis that macrophages may express matrix components now requires further studies to track tagged matrix components, which would allow more detailed investigation of the underlying mechanisms of matrix assembly and repair in the kidney. We imply that the isolated macrophages at steady state express matrix components, and that macrophages may localise to collagen-expressing areas within the kidneys. Whether macrophages secrete matrix components to facilitate basement membrane assembly, disassembly, turnover and organisation is beyond the scope of this paper but intended in our future studies. Fluorophore tagging of matrix components has been used to observe matrix dynamics in *C.elegans* [69] and *Drosophila* [58]. The development of these tools in mice with dendra2-tagged laminin beta-1 [70] and labelled nidogen [71] will enable more extensive study of basement membrane secretion and the cell types involved.

In summary, we segregated three macrophage subsets defined by CX_3_CR1 and CD81 and determined their dependence on monocyte replenishment. Although transcript expression from subsets indicates potential non-myeloid contribution, the identification of matrix expression within kidney macrophages may indicate a potential role in matrix regulation, prompting further work to elucidate this function within the kidney microenvironment.

## Methods

### Mice

C57BL/6 J (CD45.2) male mice (Envigo, UK) were housed at The University of Manchester Animal Unit under SPF conditions. Congenic CD45.1 were backcrossed with C57BL/6 J for at least 10 generations to generate CD45.1/2 mice for chimeric experiments. *Cx3cr1*^CreER^ mice [24] bred inhouse were crossed with *R26-yfp* mice [29] (both from Jackson Laboratory, USA). Mice aged 10–12 weeks were used unless stated otherwise. Experiments were approved by The University of Manchester Local Review Committee and carried in accordance with the UK Home Office Animals (Scientific Procedures) Act 1986.

### Tamoxifen treatment

Tamoxifen (Sigma-Aldrich) was dissolved in 10 % ethanol and 90 % corn oil and administered by oral gavage for 5 consecutive days to *Cx3cr1*^CreER^ X *R26-yfp* mice aged 7 weeks old [30]. Kidneys were harvested 1 and 16 weeks post-tamoxifen.

### Shielded chimeras

Host C57BL/6 (CD45.2) male mice aged 10–12 weeks were anaesthetized by intraperitoneal administration of 80 mg/kg ketamine (Ventoquinol) and 8 mg/kg xylazine (Bayer) as previously described, placed beneath a lead sheet shielding the lower two thirds of the body and irradiated with a split dose of sublethal irradiation (2 × 5.5 Gy) [30]. After recovery from anesthesia, each mouse was reconstituted with an intravenous injection of 2 × 10^6^ bone marrow cells from donor animals (CD45.1/2). Bone marrow cells were depleted of CD90.2 ^+^ *T* cells using CD90.2 beads (Miltenyi Biotec). Mice received 0.03 % enrofloxacin in drinking water in the week prior to irradiation and for 3 weeks post-irradiation, and housed in autoclaved cages with sterile bedding, water and diet. The minimum reconstitution time was 7 weeks.

### Kidney leukocyte isolation

The kidney capsules were removed, and tissues minced, followed by digestion at 37 °C for 30 min at 150 rpm in Hank’s Balanced salt solution with calcium chloride and magnesium sulphate containing 0.1 mg/ml Liberase TM Research Grade enzyme (05,401,127,001, Roche) and 0.1 mg/ml DNaseI (11,284,932,001, Roche). Digested tissue was passed through a 40 μm strainer and resuspended in Red Blood Cell (RBC) lysing buffer (Hybri-Max™) for 3 min at room temperature. Cells were enriched with CD45 microbeads (Miltenyi Biotec) and resuspended in PBS prior to flow staining and FACS.

### Whole blood preparation

500μl blood was collected into EDTA-coated syringes through cardiac puncture from mice. Suspensions were mixed with RBC lysing buffer for 3 min on ice twice and resuspended in PBS until flow staining.

### Lung leukocyte isolation

Minced lung tissue was digested in the same way as the kidneys above. Digested tissue was passed through a 40 μm strainer, resuspended in Red Blood Cell (RBC) lysing buffer (Hybri-Max™) for 3 min and resuspended in PBS prior to flow staining.

### Flow and imaging cytometry

Single cell suspensions (2.5 × 10^5^ – 2.0 × 10^6^ total cells) from the kidney and blood were washed with PBS and stained with the Zombie UV™ Fixable Viability Kit (Biolegend) to exclude non-viable cells. Subsequently, cells were stained with fluorochrome-conjugated extra-cellular targets for flow (Supplemental Table 8A) and imaging (Supplemental Table 8B) cytometry containing anti-CD16/CD32 (2.4G2, BD Biosciences) diluted in flow buffer containing PBS and 2 % (v/v) FCS at 4°C for 15 min. Cells were washed with flow buffer and acquired live or fixed in 1 % paraformaldehyde (Sigma-Aldrich) at room temperature for 10 min and washed with flow buffer before acquisition. Cell acquisition for flow cytometry was performed on the BD LSRFortessa™ with BD FACSDiva™ software (BD Biosciences) and data analyzed using FlowJo v10 (Tree Star). Cell acquisition for imaging cytometry was performed on the ImageStream^®X^ Mark II imaging flow cytometer (Merck Millipore) and data analyzed using the IDEAS software version 6 (Merck Millipore).

### Mouse kidney macrophage and blood monocyte isolation by FACS

Single-cell suspensions prepared for FACS as above with modifications: different antibodies (Supplemental Table 8C) were used, and suspensions were stained with 0.25 μg/ml of DAPI prior to acquisition to exclude non-viable cells. Leucocytes were enriched prior to sorting and cell purity was >85 % as defined by flow cytometry for CD45 antibody (clone 30-F11, Biolegend). Kidney macrophages (DAPI^−^CD45^+^Lin^−^Ly6C^−^CD64^+^MHCII^+^ and CX_3_CR1^−^CD81^−^ P1, CX_3_CR1^+^CD81^−^ P2 or CX_3_CR1^+^CD81^+^ P3) and blood monocytes (DAPI^−^CD45^+^Lin^−^CD115^+^CD11b^+^Ly6C^+^MHCII^−^) were sorted using the BD Influx™ (BD Biosciences) using the 140 μm nozzle and 7.5 psi pressure, to a purity of 95–99 % as described previously [72]. Cells were sorted directly into RLT buffer (QIAGEN) containing 0.1 % (v/v) β-mercaptoethanol and stored at –80 °C for RNA extraction. For matrix enrichment, cells were incubated in ice-cold buffer solution (10 mM Tris, 150 mM sodium chloride, 25 mM EDTA, 1 % (v/v) Triton X-100) at 4 °C overnight.

### RNA extraction

RNA was extracted from 1.5 – 2.0 × 10^5^ cells per population (Ly6C^+^ blood monocytes, P1, P2 and P3 kidney macrophages) for each independent sort from the kidneys and blood of 4–5 pooled C57/BL6 mice at steady state using the RNeasy micro kit (QIAGEN). Quality control of the RNA sample for all cell populations was assessed using the RNA ScreenTape 2200 Tapestation assay and system (Agilent Technologies) and quantified using a Qubit 2.0 Fluorimeter (ThermoFisher Scientific).

### Bulk RNA-sequencing and analyses

Libraries were prepared with the SMART-Seq v4 Kit (Takara Bio). Unmapped paired-end sequences from an Illumina HiSeq4000 sequencer were tested by FastQC (Babraham Bioinformatics). Sequence adaptors were removed, and reads were quality trimmed using Trim-momatic_0.39 [73]. Bulk RNA-sequencing data are available from the ArrayExpress repository. The reads were mapped against the reference mouse genome (mm10/GRCm38) and counts per gene were calculated using annotation from GENCODE M25 (http://www.gencodegenes.org/) using STAR_2.7.7a [74]. Normalisation, principal component analysis, and differential expression was calculated with DESeq2_1.18.1 [75]. Adjusted *p*-values were corrected for multiple testing (Benjamini and Hochberg method). Heatmaps were drawn with complexHeatmap v2.16.0 [76] Gene ontology enrichment was studied using clusterProfiler v4.8.3 [77] and ReactomePA 1.44.0 [78].

Accession E-MTAB-11,525: https://www.ebi.ac.uk/biostudies/arrayexpress/studies/E-MTAB-11525?key=482fb394–1c6a-4f7f-b685–14bf58455944.

### Bulk gene expression deconvolution

To determine the cell composition in our bulk RNA-sequencing data, single-cell RNA-sequencing (scRNA-seq) data from GSE128993 [18], GSE140023 [6], GSE174324 [38] and GSE193892 [25] were obtained from the Gene Expression Omnibus (GEO; https://www.ncbi.nlm.nih.gov/geo/) database. scRNA-seq data were processed using various packages in R (v4.1). Briefly, low quality cells identified using a combination of median absolute deviation (MAD), as implemented by the “isOutlier” function in the scuttle R package (v1.4.0) and exacted thresholds were removed before data integration. The log-normalized expression values of the combined data were computed using the “multiBatchNorm” function from the batchelor R package (v1.10.0). The per-gene variance of the log-expression profile was modelled using the “modelGeneVarByPoisson” function from the scran R package (v1.22.1) and top 2000 highly variable genes were selected. Mutual nearest neighbors (MNN) approach available from the batchelor R package was used to perform batch correction. Then, first 20 dimensions of the MNN data were used as input to produce the t-stochastic neighbour embedding (t-SNE) projection and uniform manifold approximation and projection (UMAP) using the “runTSNE” and “runUMAP” functions from the scater R package (v1.22.0) respectively. 30 non-doublet cell clusters (from 18 superclusters) were identified using Leiden clustering algorithm from the igraph R package (v1.5.1). Cell clusters were annotated using known marker genes and that provided by the authors in the corresponding publications.

A third of the cells (*N =* 8753) from the combined scRNA-seq data was randomly selected as training cells, and their count data was used to build the mouse kidney signature matrix on the CIBERSORTx website (https://cibersortx.stanford.edu/). The remaining cells (as test cells) were used to generate pseudobulk samples using the “aggregateA-crossCells” function from the scuttle R package by aggregating cells (1) of the same GEO accession, and (2) randomly divided all test cells into 40:60 ratio (*N =* 7002 and 10,504). These pseudobulk samples were used to validate the proportions of cell types estimated by CIBERSORTx (Supplementary Figure S4c, Supplementary Table S3b). Cell fraction imputation was performed on the CIBERSORTx website with S-mode (single cell mode) batch correction enabled and at 100 permutations for statistical analysis.

### Sample preparation for proteomics

Sample enrichment for ECM proteins was performed as previously described [39,40]. Sorted cells were lysed with an extraction buffer (10 mM Tris pH 8.0, 150 mM NaCl, 25 mM EDTA, 1 % (v/v) Triton X-100, protease inhibitor (11,873,580,001, Roche)) overnight at 4 °C, then centrifuged at 14,000 × *g* for 10 min at 4 °C to yield fraction 1 (supernatant). The pellet was incubated with 20 mM Na_4_OH and 0.5 % (v/v) Triton X-100 in 0.1 M PBS for 1 hour at 4 °C. After centrifugation, the supernatant (fraction 2) was combined with fraction 1 (1:1) into a soluble fraction, and the pellet was resuspended in a lysis buffer (5 % (v/v) SDS, 50 mM triethylammonium bicarbonate (TEAB) pH 7.5) and sonicated using a Covaris LE220+ Focused Ultrasonicator (Covaris). After centrifugation, the supernatant (insoluble fraction) was collected. Sample fractions were reduced with 5 mM dithiothreitol (DTT) for 10 min at 60 °C and alkylated with 15 mM iodoacetamide for 30 min in the dark. 5 mM DTT was further added to quench residual alkylation reaction. Samples were acidified with 1.2 % (v/v) phosphoric acid then added with a methanolic solution containing 100 mM TEAB (pH 7.1) and transferred to a 96-well S-Trap™ plate (Protifi). After centrifugation at 1000 × *g* for 2 min, proteins were digested with 0.16μg sequencing grade modified trypsin (V5111, Promega) for 1 hour at 47 °C. The peptides were then washed with 0.1 % aqueous formic acid and eluted from the plate by centrifugation with 0.1 % (v/v) formic acid in 30 % (v/v) acetonitrile. After desalting using oligo R3 POROS beads (Thermo Fisher Scientific), peptides were collected and dried to completeness by vacuum centrifugation.

### Mass spectrometry data acquisition and analysis

Peptide sample fractions were analyzed by liquid chromatographytandem mass spectrometry using a Q Exactive™ Plus Hybrid Quadrupole-Orbitrap™ Mass Spectrometer (Thermo Fisher Scientific) mass spectrometer. Peptides were selected for fragmentation automatically by data-dependent analysis. Raw spectra data were analyzed using MaxQuant [79], and peaks were searched against the mouse SwissProt and TrEMBL databases (v.2022). Carbamidomethylation of cysteine was set as fixed modification, and oxidation of methionine, proline and lysine, and N-terminal acetylation as variable modifications. Only tryptic peptides with up to 2 missed cleavages were considered for the search. Relative abundances were determined by label-free quantification based on precursor ion intensity. The mass spectrometry proteomics data have been deposited to the ProteomeXchange Consortium via the PRIDE partner repository [80] with the dataset identifiers PXD032301. To identify ECM and basement membrane components, the final protein list was cross-referenced with annotate data from the human matrisome [35] and basement membraneBASE (https://bmbasedb.manchester.ac.uk/). A specific list of markers (Supplementary Table S9) was selected to search the proteomics data for contamination by non-immune cells (podocytes, endothelial cells, mesangial cells, fibroblasts and tubular epithelial cells) using R studio.

### Functional enrichment analyses

Functional enrichment analysis was performed on both transcriptomic and proteomic datasets in R (version 4.1.1) using the package ClusterProfiler (version 4.0.3) [77] with keyType = “EMSEMBL” and ReactomePA [78]. In the case of transcriptomic data, significantly differentially expressed genes from P1, P2 and P3 kidney macrophages against blood monocytes (adjusted *p*<0.05) were used against the mouse genome as background. The reads were mapped against the reference mouse genome (mm10/GRCm38) and counts per gene were calculated using annotation from GENCODE M25 (http://www.gencodegenes.org/) using STAR_2.7.7a [74]. Normalisation, Principal Components Analysis, and differential expression was calculated with DESeq2_1.18.1 [75]. Adjusted *p*-values were corrected for multiple testing (Benjamini and Hochberg method). Heatmaps were drawn with complexHeatmap v2.16.0 [76]. Gene enrichment was studied using ReactomePA 1.44.0 [78] (PMID:26,661,513). (depending on what we use). In the case of the proteomic datasets, identified proteins lists were used against a constructed kidney background to be representative of those proteins identifiable by mass spectrometry for both soluble and less soluble matrix fractions. The kidney background protein lists were curated from kidney datasets (PXD022363, PXD025874, PXD025911) obtained from PRIDE [80] and one unpublished dataset (Lausecker, Lennon, personal communication). All enrichment plots were generated using the enrichplot package.

### Immunofluorescence

Kidney cryosections, 3 μm thick, were permeabilized with PBS containing 2 % (v/v) BSA, 1 % (v/v) donkey serum and 0.1 % (v/v) Triton X-100 pH 8.0 prior to incubation with primary antibody (Supplemental Table 10A) at 4 °C overnight. Sections were washed with PBS and incubated with secondary antibodies (Supplemental Table 10B) at room temperature for 45 min. Sections were subsequently stained with Hoechst 33,342 (Thermo Fisher Scientific) at room temperature for 10 min. Slides were mounted with Prolong Gold Antifade Mountant (Life Technologies). Sections were viewed on a Zeiss Axio Imager.D2 upright microscope and images acquired using a Coolsnap HQ2 camera (Photometrics) through Micromanager software v1.4.23 prior to analyses on ImageJ (http://imagej.net/Fiji/Downloads).

### RNAScope in situ hybridization

RNAscope in situ hybridization was performed on 4um FFPE kidney section using an RNAscope multiplex fluorescent reagent kit v2 (Advanced Cell Diagnostics, Newark, CA, USA) and a TSA plus system (Akoya Biosciences, Marlborough, MA, USA) The target probes used are: Mm-Wt1-C1, Mm-Lama5-C2, Mm-Col4a1-C3, Mm-Adgre1(F4/80)-C4. Briefly, paraffin sections were baked for 1 h at 60 °C, deparaffinized, subjected to hydrogen peroxide for 10 min at room temperature, boiled in target retrieval buffer for 15 min, and treated with protease plus for 30 min at 40 °C. The sections were then hybridized with the target probes for 2 h at 40 °C, followed by signal amplification reactions and fluorescence (Opal 520, 570, 620 and 690) detection according to the manufacturer’s protocols. Confocal microscopy was performed on RNAscope labelled samples using a Zeiss LSM880and the ZEN 2.1 SP3 FP2 software (Zeiss, Oberkochen, Germany). Images were captured using a 40x NA1.4 oil immersion objective with a pinhole <3 airy units. An argon-ion laser producing a 488 nm beam, was used to excite Opal-520 and Opal-570 dyes and the emission collected using a λ spectral scan spanning 500–620 nm. The image was then linear unmixed to separate the two dyes. For the remaining fluorescent dyes, images were taken sequentially. DAPI was excited using a 405 nm diode laser, Opal-620 with a 594 nm Helium-Neon laser and Opal-690 with 633 nm Helium-Neon laser. Appropriate emission spectra (~30–70 nm around peak emission) were collected using a Quasar detector array. Images were acquired at 8-bit and at 1024×1024 pixel resolution.

### Statistical analyses

Previous successful experiments were used [28,30,33] to determine animal group size and steps taken to ensure the minimum number of mice to allow robust and biologically relevant results to be acquired. Comparisons between two unpaired, non-normally distributed data-points were carried out using the Mann-Whitney U test. Comparisons between multiple groups were performed with a Kruskal-Wallis test. Statistical analyses and comparisons were performed on GraphPad Prism version 9.0 (GraphPad, USA) unless stated otherwise. For the proteomic data, peptide intensities were log2-transformed and normalized by median centering, and decoy and contaminant peptides were filtered out. Statistical comparison was carried out using the msqRob2 package for R [81–83].

## Supplementary Material

Supplementary material associated with this article can be found, in the online version, at doi:10.1016/j.matbio.2024.02.002.

Supplementary Material

## Data Availability

Data will be made available on request.
