# Supplementary material for: Kidney resident macrophages have distinct subsets and multifunctional roles
Source: Matrix Biol. Author manuscript; Available in PMC 2026 Aug 14. (PMC7619362; doi:10.1016/j.matbio.2024.02.002)
Supplement: Supplementary file 1 [file EMS216253-supplement-Supplementary_Material.docx]

**Supplementary Material for**

**Kidney resident macrophages have distinct subsets and multifunctional roles.**

^#^Christine Chew^1,2^, Oliver J Brand^1^, Tomohiko Yamamura^2^, Craig Lawless^2^, Mychel Raony Paiva Teixeira Morais^2^, Leo Zeef^3^, I-Hsuan Lin^3^_,_ Gareth Howell^1^, Sylvia Lui^1^, Franziska Lausecker^2^, Christopher Jagger^1^, Tovah N Shaw^4^, Siddharth Krishnan^1^, Flora A McClure^1^, Hayley Bridgeman^1^, Kelly Wemyss^1^, Joanne E Konkel^1^, *Tracy Hussell^1^, *Rachel Lennon^2,5^.

**Author affiliations**

1. Lydia Becker Institute for Immunology and Inflammation, Division of Infection, Immunity and Respiratory Medicine, School of Biological Sciences, Faculty of Biology Medicine and Health, The University of Manchester, M13 9PL, UK.

2. Wellcome Centre for Cell-Matrix Research, Division of Cell-Matrix Biology and Regenerative Medicine, School of Biological Sciences, Faculty of Biology Medicine and Health, The University of Manchester, Manchester Academic Health Science Centre, Manchester, M13 9PT, UK.

3. Bioinformatics Core Facility, Faculty of Biology Medicine and Health, The University of Manchester, M13 9PL, UK

4. Institute of Immunology and Infection Research, School of Biological Sciences, University of Edinburgh, Ashworth Laboratories, Edinburgh, EH9 3FL, UK.

5. Department of Paediatric Nephrology, Royal Manchester Children’s Hospital, Manchester University Hospitals NHS Foundation Trust, Manchester Academic Health Science Centre, Manchester, M13 9WL, UK.

^#^First author, *corresponding author

# Supplementary Figures

**Supplementary Figure S1. CX_3_CR1 and CD81 identify three kidney macrophage subsets. a,** Representative flow cytometry contour plot (first on the left) and histograms (three graphs on the right) verifying CX_3_CR1, CD81 and CD74 expression in MP1 and MP2 kidney macrophages (grey: FMO, purple: MP1, green: MP2). **b,** Representative flow cytometry contour plots assessing CX_3_CR1 and CD81 expression in monocytes (Ly6C^+^MHCII^-^, Ly6C^+^MHCII^+^) and the macrophage pool (Ly6C^-^MHCII^+^). **c,** Representative flow cytometry contour plot showing the back-gating of P1, P2 and P3 kidney macrophage subsets onto CD11b versus F4/80. Flow plots and histograms are representative of at least > 6 experiments. **d,** Imaging cytometry panels (top) and histograms (bottom) of kidney macrophage subsets defined by CX_3_CR1 and CD81 in C57BL/6 mice at steady state. Kidney single cell suspensions were incubated with antibodies for a viability dye, CD45, lineage (CD3, CD19, Gr-1), CD64, MHCII, CX_3_CR1, CD81 and F4/80 antibodies and macrophages identified based on the expression of these markers, which include the P1, P2 and P3 kidney macrophage subsets. **e,** Immunofluorescence of F4/80 and CD81 (macrophage markers, purple), pan-collagen IV (PanColIV) and Col4a2 (basement membrane markers, green) and podocyte marker (WT1, red) in kidney sections (3μm thick) from C57BL/6 mice at steady state. Merge: White scale bar, 100μm. Images are representative of >2 independent experiments.

**Supplementary Figure S2. CX_3_CR1 and CD81 identify kidney macrophages with distinct monocyte replenishment. a,** *Cx3cr1*^CreER^ X *R26-yfp* reporter mouse model and the assessment of macrophage replenishment by blood monocytes represented by YFP^-^ cells 1 weeks after tamoxifen treatment. Representative flow cytometry contour plots (left) to assess YFP expression in P1, P2 and P3 (top left) and MP1 and MP2 (bottom left) kidney macrophage subsets. Graph (right) showing frequencies of YFP-expressing macrophage subsets defined by the new (P1, P2, P3) and conventional (MP1, MP2) gate. **b,** C57BL/6 (CD45.2) mice irradiated with the lower third of the body shielded from irradiation. These mice received two doses of 5.5Gy irradiation followed by donor CD45.1/2 bone marrow cells on the same day and left to reconstitute for at least 7 weeks prior to kidney and blood harvest. **c,** Total number of cells from the kidney macrophage pool (left) and P1, P2 and P3 macrophage subsets (right) in non-irradiated C57BL/6 and shielded chimeras. **d,** Representative flow cytometry contour plots showing the percentage of chimerism for MP1 and MP2 macrophages subsets from the kidneys of shielded chimeras. Data are representative of two independent experiments and graphs show the median (IQR) of n=4-6 mice per group. Statistical analyses between two groups were performed with Mann Whitney U test and multiple comparisons with a Kruskal-Wallis test.

**Supplementary Figure S3. a,** Hierarchical cluster analyses of genes in Ly6C^+^ monocytes and kidney macrophages (P1, P2 and P3) from bulk RNA-sequencing analyses represented in a heatmap. **b,** Representative flow cytometry histograms showing the expression of MerTK, Axl and CD64 (encoded by *Fcgr1*) by F4/80^+^ kidney and lung macrophages (grey: fluorescence minus one (FMO) control, turquoise: full stain). Histogram data are representative of n=4 samples from one experiment, **c,** Reactome enrichment analyses of transcriptomic datasets from bulk RNA-sequencing from the macrophage pool versus Ly6C^+^ monocytes, P2 versus P1, P3 versus P1 and P3 versus P2 macrophages. Data (**a, c**) are representative of three independent cell sorts from the kidneys and blood of 4-5 pooled C57BL/6 mice at steady state.

**Supplementary Figure S4. a,** Scatterplots showing pairwise comparison of the average gene expression intensities (RPKM; reads per kilobase per million mapped reads) of P1, P2 and P3 macrophage subsets and Ly6C^+^ monocytes versus bulk RNA-sequencing data from 3 month old mouse kidneys, and P1-3 subsets against Ly6C^+^ monocytes. Macrophage gene markers are coloured in red, other immune markers in orange and non-immune markers in blue. These include: *C1g* and *Ly6c2* (macrophage), *Ms4a1* (B cell), *Cd3d* (T cell), *Nkg7* (natural killer cell), *Cd209a* and *Itgae* (classical dendritic cell), *Nphs2* and *Podxl* (podocyte), *Lrp2* and *Maf* (proximal tubule), *Umod* and *Slc12a1* (loop of Henle), *Aqp2* and *Atp6v1g3* (collecting duct), and *Col3a1* (stromal cell). Dotted diagonal line denotes X=Y. **b,** Stacked barplot depicting the proportion of the 16 cell types in Ly6C^+^ monocytes and P1-3 subsets predicted by CIBERSORTx. **c,** Stacked barplot depicting the proportion of the 16 cell types in the pseudobulk sample from the actual scRNA-seq analysis and that predicted by CIBERSORTx. Number of cells that made up the pseudobulk samples are shown in round brackets. References to datasets in the main manuscript text.

**Supplementary Figure S5. ECM components detected in kidney macrophages. a,** Representative flow cytometry contour plots showing the gating strategy for identifying the kidney macrophage pool defined by CD45^+^CD3^-^CD19^+^Ly6G^-^Ly6C^-^CD64^+^MHCII^+^. Cells were identified as (I) Side scatter area (SSC-A) versus forward scatter area (FSC-A), (II) forward scatter height (FSC-H) versus FSC-A to exclude cell doublets (figure legend continued on the following page), (III) Live/dead versus FSC-A to identify live or viable cells (IV) Lineage (Lin: CD3, CD19, Ly6G, Ly6C) versus CD45 to identify hematopoietic populations and exclude lymphoid and neutrophil populations, (V) CD64 versus MHCII to identify the macrophage pool. **b,** Soluble and less soluble fractions prepared from the kidney macrophage pool (n=3) analyzed by mass spectrometry and the overlap for the total number of proteins represented by a Venn diagram. **c,** Dot plot depicting selected functional enrichment pathways of proteomic datasets from mass spectrometry analyses showing biological processes and reactome of the soluble (top line) and less soluble (bottom line) fraction of the kidney macrophage pool. **d,** RNAscope of F4/80 (red), Col4a1 (orange), Wt1 (green), Lama5 (yellow) and DAPI (blue) in kidney sections from C57BL/6 and *Col4a5^-/-^* (Col4a5 KO) mice.

# Supplementary Figure S6. a, Representative flow cytometry plots showing purity check on the CD45^+^DAPI^+^Lin^+^ cells, b, Compensation matrix panel for kidney macrophage flow cytometry analyses.

**Supplementary Table Legends**

**Tables on Figshare:** <https://figshare.com/s/b3f66cf632bd482a9d65>

**Supplementary Table S1.** Kidney macrophage subsets defined by CX_3_CR1 and CD81 compared to subsets defined in previous publications in mice.

**Supplementary Table S2.** Functional enrichment analyses of pathways performed on bulk RNA-seq datasets (FDR adjusted p<0.05) showing biological processes, molecular function, cellular components and reactome of kidney macrophage subsets **a,** P1, **b,** P2 and **c,** P3 versus Ly6C^+^ monocytes.

**Supplementary Table S3. a,** Tables for scatterplots of podocyte marker gene expression in P1 (left), P2 (middle) and P3 (right) subsets versus bulk RNA-sequencing data from 3 month old mouse kidneys. **b,** Tables for the proportion of transcripts from different cell types in Ly6C^+^ monocytes, P1, P2 and P3 subsets from CIBERSORTx analyses of scRNA-sequencing datasets. References to datasets in the main manuscript text.

**Supplementary Table S4.** Proteomic datasets of all proteins identified in the **a,** less soluble, **b,** soluble and **c,** ECM fraction of the kidney macrophage pool.

**Supplementary Table S5.** Functional enrichment analyses of pathways (biological processes, molecular function, cellular components and reactome) performed by comparing kidney background protein lists curated from kidney datasets (detailed in the methods section) to proteomics datasets from the **a,** cellular and **b,** ECM fraction of the isolated kidney macrophage pool.

**Supplementary Table S6.** Proteomic dataset of all proteins identified in the soluble fraction of the kidney macrophage pool from wild type and *Col4a5^-/-^* mice.

**Supplementary Table S7.** List of specific markers indicating possible contamination from non-myeloid cells within the proteomic dataset of the kidney macrophages pool from wild type mice: **a,** soluble fraction, **b,** ECM fraction

**Supplementary Table S8. a,** List of fluorochrome-conjugated antibodies for flow cytometry. **b,** List of fluorochrome-conjugated antibodies for imaging cytometry. **c,** List of fluorochrome-conjugated antibodies for FACS of kidney macrophage and blood monocyte isolation.

**Supplementary Table S9.** List of non-myeloid cell markers to assess for cell contamination within the kidney macrophage pool isolated for proteomics analyses.

**Supplementary Table S10.** List of antibodies for immunofluorescence. **a,** Primary antibodies, **b,** Secondary antibodies, **c.** RNA probes for RNAscope**.**
